# Point-of-Care Ultrasound Findings of Ovarian Hyperstimulation Syndrome: A Case Report

**DOI:** 10.5070/M5.52324

**Published:** 2026-10-31

**Authors:** Terra Thimm, Roger Tillotson, Joseph Minardi, Anthony Steratore

**Affiliations:** * West Virginia University School of Medicine, Department of Emergency Medicine, Morgantown, WV; * David and JoAnn Shaw Center for Simulation Training and Education for Patient Safety, West Virginia University, Morgantown, WV

## Abstract

Ovarian hyperstimulation syndrome (OHSS) is a rare but potentially life-threatening complication of assisted reproductive technology (ART). Prompt recognition and management are essential to reduce morbidity.^1^ This case describes a 30-year-old woman undergoing ART who presented to the emergency department (ED) with abdominal pain, distention, lightheadedness, nausea, vomiting, and shortness of breath following transvaginal oocyte retrieval. Physical examination revealed a tense, diffusely tender abdomen, and point-of-care ultrasound (POCUS) demonstrated enlarged ovaries bilaterally with multiple follicles and moderate peritoneal free fluid, confirming the diagnosis of OHSS. Laboratory findings included leukocytosis, hemoconcentration, and hypoproteinemia. The patient received supportive care with intravenous fluids and analgesia and, during admission, underwent therapeutic paracentesis, resulting in the removal of roughly three liters of ascitic fluid. Her symptoms resolved, and she was discharged on hospital day three with complete recovery noted at follow-up. This case highlights the diagnostic value of POCUS in the emergency setting for early identification of OHSS. As the use of ART increases, emergency clinicians should be aware of OHSS presentations and consider bedside ultrasound as a key diagnostic tool to expedite care and improve outcomes.

**Topics:**

Assisted reproductive technology, point of care ultrasound, ovarian hyperstimulation syndrome.

## Introduction

Ovarian hyperstimulation syndrome is a rare and potentially life-threatening iatrogenic complication of controlled ovarian stimulation (COS) during ART cycles. The pathophysiology of OHSS is largely attributed to an increase in vascular permeability of the ovarian and peritoneal capillaries secondary to ovarian hypersecretion of vascular endothelial growth factor (VEGF).^1^ Ovarian hyperstimulation syndrome is characterized by massive ovarian enlargement and acute third-space fluid shifts, which can result in intravascular hypovolemia, hemoconcentration, and electrolyte imbalances.^2^–^4^ Hemoconcentration, as well as hypercoagulation secondary to elevated serum estrogen concentrations, can result in venous or arterial thrombosis, which may lead to permanent injury or death.^5^ Other complications of OHSS include abdominal compartment syndrome, renal dysfunction, ovarian torsion, acute respiratory distress syndrome, and hemodynamic instability.^5^ Data on its prevalence as an emergency department presentation is sparse, however this condition occurs in up to 30% of all ART cycles, with severe OHSS occurring in 1–3% of cycles.^1^,^6^ Here we present a case of severe ovarian hyperstimulation syndrome presenting with abdominal pain that was immediately identified by the emergency physician utilizing point-of-care ultrasound (POCUS).

## Presenting concerns and clinical findings

A 30-year-old, G3P0030, with a history of anovulatory PCOS and undergoing ART, presented to our ED with five days of abdominal pain and distention, lightheadedness, nausea, vomiting, and shortness of breath. Her symptoms began and worsened after ultrasound-guided transvaginal oocyte retrieval five days prior. Prior to oocyte retrieval she was self-administering follicle stimulating hormone [Follistim] 150IU and human menopausal gonadotropin [Menopur] 75IU injections for ovarian stimulation, and two days prior to retrieval she administered a trigger injection of hCG 10,000IU in accordance with her treatment protocol. Her past medical history was significant for two prior ectopic pregnancies resulting in bilateral salpingectomies. Her ED vital signs were significant for blood pressure of 148/91 millimeters of mercury and heart rate of 119 beats per minute. On general physical exam, she was acutely ill appearing, in moderate distress secondary to abdominal pain. Abdominal exam was significant for diffusely tender and tense abdomen with rebound and guarding.

## Significant findings

Transabdominal POCUS in the right upper quadrant revealed clearly delineated pockets, the hypoechoic appearance being consistent with ascites, surrounding the liver and the gallbladder (Figure 1). A view of the left upper quadrant revealed a similar hypoechoic fluid collection that filled the space below the diaphragm and surrounded the spleen (Figure 2). A transabdominal pelvic view revealed enlarged ovaries, identifiable by the numerous, large, and well-delineated hypoechoic collections within that represent the enlarged follicles (Figure 3). The extent of the hypoechoic free fluid collections and the enlargement of the ovaries and their follicles is more apparent when viewed dynamically (Video).

## Patient course

Labs were significant for a white blood cell count of 25.8k/μL, hematocrit of 42.7%, sodium of 133 mEq/L, and hypoproteinemia. She was treated with intravenous morphine, ondansetron, one liter normal saline, and admitted to the gynecology service. She underwent ultrasound guided paracentesis on day one of admission with 3.2 liters of fluid drained, after which her symptoms resolved. She was discharged on day three of admission. She was seen in clinic two days after discharge and noted to be doing well.

## Discussion

Prior to the initiation of ovarian stimulation, women at high risk of developing OHSS should be identified by their reproductive health specialist and protocols should be selected that minimize risk of this potential complication.^5^ If OHSS prevention strategies have not been implemented or are ineffective, patients may present to the ED with abdominal pain, abdominal distention, nausea, vomiting, diarrhea, shortness of breath, or syncope. These symptoms in combination with any history of ART should warrant at least initial consideration of OHSS on the differential diagnosis for the EM practitioner. This differential diagnosis should also include ectopic pregnancy, hemorrhagic cyst, ruptured ovarian cyst, and ovarian torsion. Physical exam and POCUS help to narrow the differential and identify OHSS early.

Physical exam findings seen with OHSS vary with its severity but may include abdominal distention, fluid wave, and tenderness to palpation, lower extremity edema, hypotension, hypoxia, and heart murmur. Enlarged ovaries, numerous ovarian follicles, ascites, pericardial effusion, and pleural effusions can be visualized with POCUS. It can range from mild to critical with corresponding increases in morbidity and mortality.^1^

Ultrasound is the preferred imaging modality in evaluating of OHSS and, indeed, most major acute complications of ART.^5^ In early stages of this illness, intraperitoneal fluid may only be seen with transvaginal ultrasound, as fluid can be masked by enlarged ovaries with transabdominal ultrasound. As OHSS becomes more severe, ascites is more easily seen with transabdominal ultrasound.^8^,^9^ While formal data on sensitivity and specificity of POCUS in reaching this diagnosis is lacking, a growing number of existing case reports demonstrate that emergency physicians using POCUS can rapidly make an accurate diagnosis in these cases.^8^–^11^

Ultrasound should be considered as the primary diagnostic modality in diagnosing OHHS; with symptoms, laboratory values, secondary complications, urinary output and hemodynamics as the primary considerations in gauging severity (Table 1). Point of care ultrasound remains a crucial initial step, as failure to recognize OHSS can lead to increased morbidity and mortality by a variety of complications, especially with delayed diagnosis.^1^

Emergency management of OHSS for the EM physician varies significantly depending on the presence of numerous potential secondary pathologies (Table 1). Ovarian hyperstimulation syndrome significantly increases the risk of ovarian torsion and thromboembolisms, and increased clinical suspicion for these comorbid conditions in the presence of OHSS is strongly recommended.^12^ Supportive care like electrolyte management and fluid resuscitation should be considered for all cases of OHSS.^1^ The common OHSS symptom of vomiting, seen in this case, can lead to intravascular depletion even in the presence of third-spacing.^1^,^10^ Fluid resuscitation with normal saline, as in this case, or hypertonic saline(especially if hyponatremia is present) should still be considered despite the risk of continued third-spacing.^1^,^10^ The other sequelae of third-spacing represent the most common complications in OHHS, with the most common and diagnostic form of third-spacing in OHSS being ascites. As in other conditions, ascites can lead to significantly elevated intraabdominal pressure (IAP) and subsequent abdominal compartment syndrome.^1^ Significant distension, dyspnea, or oliguria should all lead to the consideration of elevated IAP and abdominal pressure measurements can solidify the diagnosis.^1^ While this patient did not have hard signs of IAP, concern for IAP may necessitate a paracentesis in the emergency setting to arrest the progression to abdominal compartment syndrome and improve oliguria and hemodynamic instability.^1^,^2^ Pleural and pericardial effusions should also be considered in patients with dyspnea or hypotension and managed accordingly.^1^,^11^ In all cases, early consultation with the patient’s reproductive specialist is strongly recommended.^1^,^9^ The disposition for patients suffering from OHSS is frequently admission to a reproductive specialist to monitor for and manage the various potential complications, as demonstrated by this case and the continued development of the patient’s ascites ultimately necessitating a paracentesis.^1^

As ART continues to proliferate, EM physicians will be increasingly caring for its patient population and managing its complications, like OHSS.^1^ Understanding the value of POCUS findings, in both the rapid diagnosis of OHSS and the assessment of some of its complications, will help EM physicians manage the critical stages of this complicated disease process and make more informed consultations with reproductive specialists.

## Supplementary Information









## Figures and Tables

**Figure 1:**
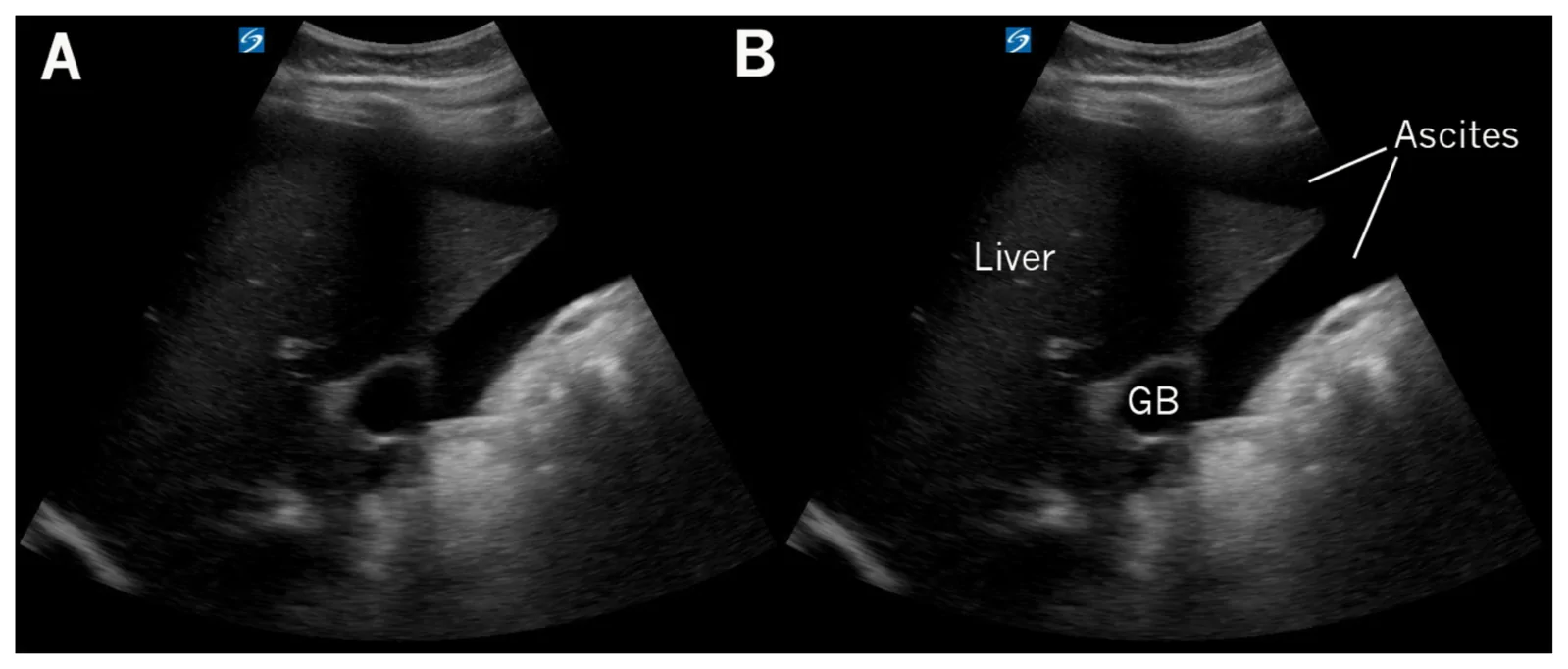


**Figure 3:**
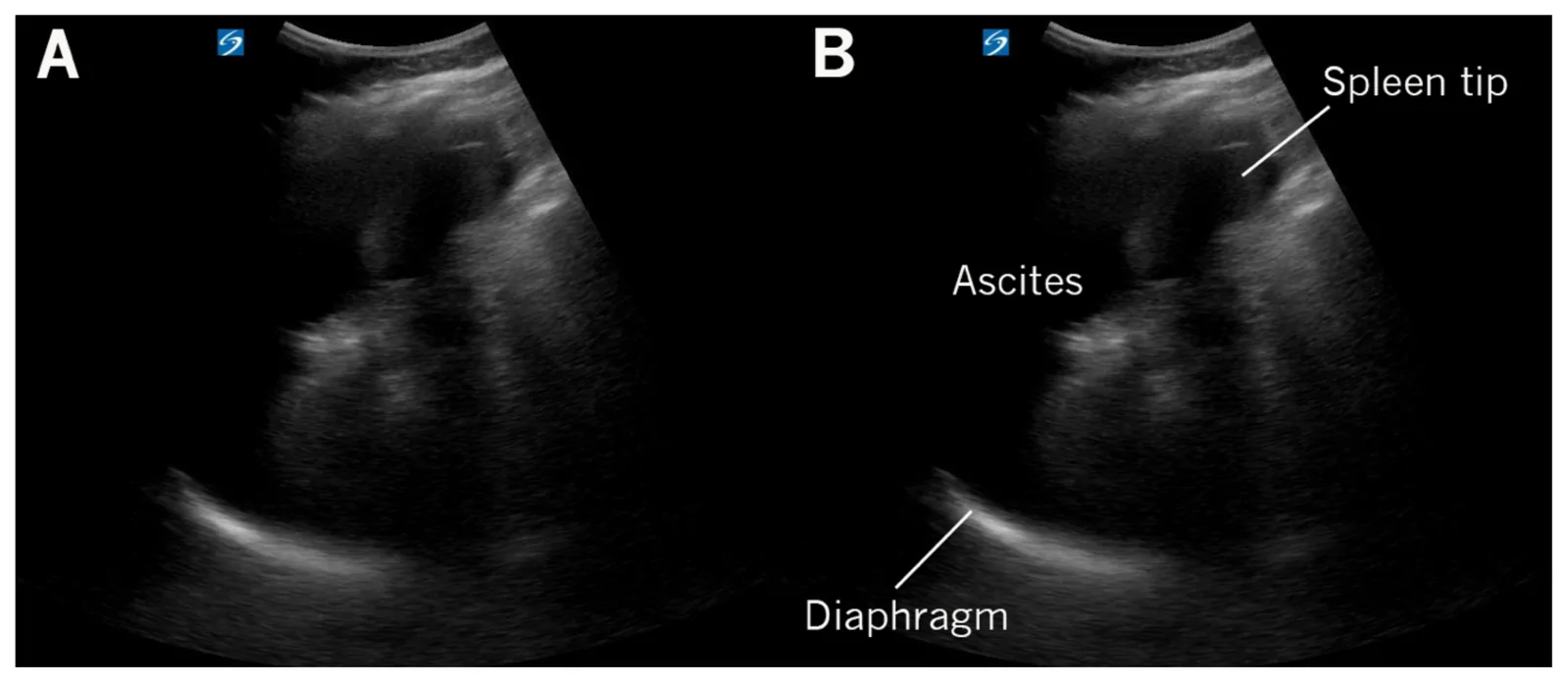


**Figure 3:**
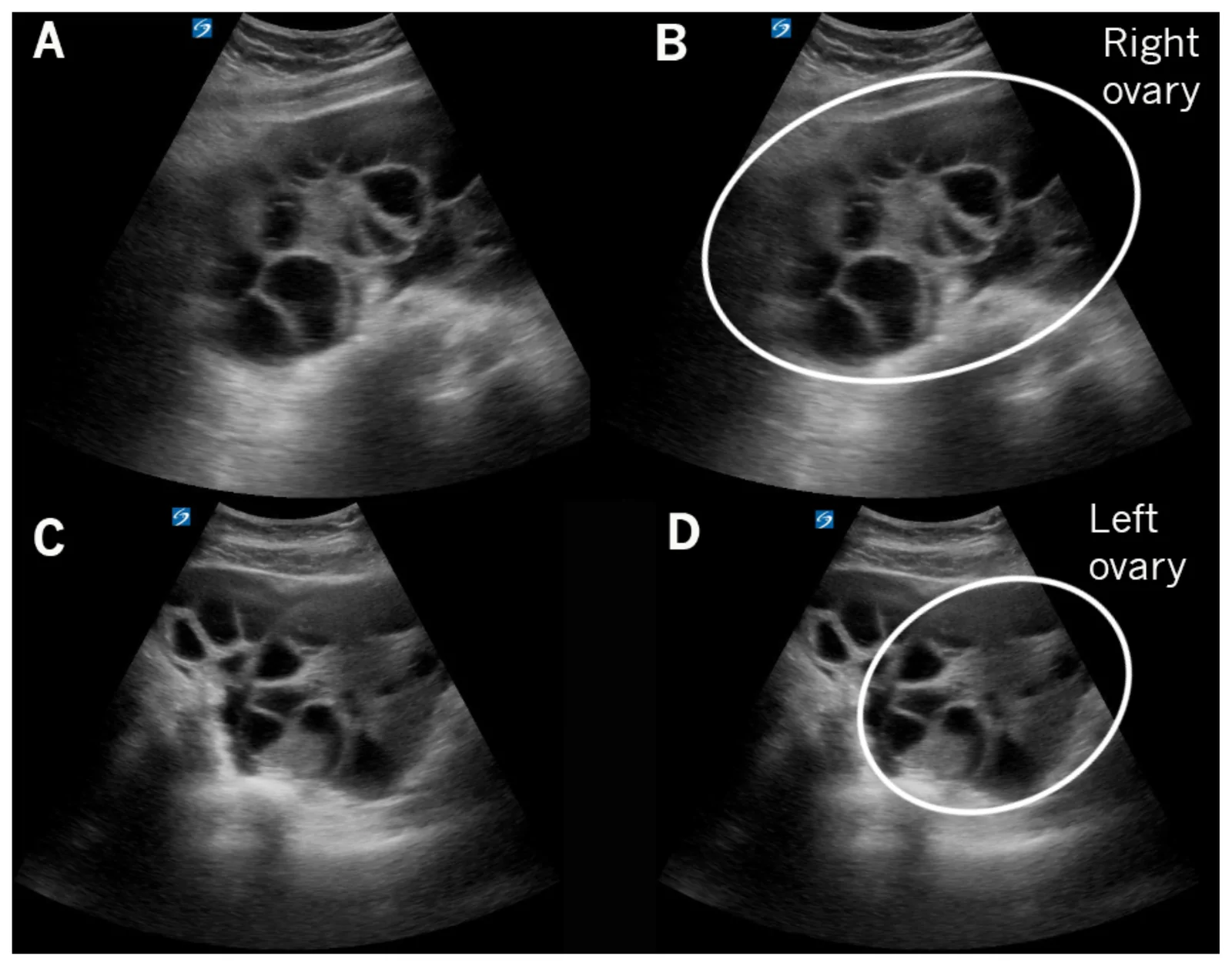
Video Link: https://youtu.be/P9Iwo6I1wtg

**Table 1:** Progression of the severity of OHSS (ARDS: acute respiratory distress syndrome; Cr: creatinine; Hct: hematocrit; K: potassium; LFTs: liver function tests; Na: sodium; OHSS: ovarian hyperstimulation syndrome; WBC: white blood cell)

Severity	Clinical Features	Laboratory Findings
Mild	Abdominal distention/discomfort, mild nausea/vomiting, diarrhea, enlarged ovaries	None
Moderate	Mild features + Ultrasonographic evidence of ascites	Hct > 41% WBC > 15,000/μL Hypoproteinemia
Severe	Mild and moderate features + Clinical evidence of ascites Severe abdominal pain Intractable nausea and vomiting Rapid weight gain (>1 kg in 24 hours) Pleural effusion Severe dyspnea Oliguria/anuria Hypotension Syncope Venous thrombosis	Hct > 55% WBC > 25,000/μL Cr > 1.6 mg/dL Cr clearance < 50 mL/min Na < 135 mEq/L K > 5 mEq/L Elevated LFTs
Critical	Severe features + Anuria/acute renal failure Arrhythmia Pericardial effusion Massive hydrothorax Thromboembolism Arterial thrombosis ARDS Sepsis	Worsening of laboratory findings seen with severe OHSS
